# Assessing the Oldness and Capacity of Radiography and Ultrasound Equipments in Tehran University of Medical Sciences

**DOI:** 10.5812/iranjradiol.6835

**Published:** 2013-08-30

**Authors:** Payman Salamati, Hossein Ghanaati, Shahram Ghasemzadeh, Amir Hossein Jalali

**Affiliations:** 1 Advanced Diagnostic and Interventional Radiology Research Center (ADIR), Tehran University of Medical Sciences, Tehran, Iran; 2 Baqiyatallah University of Medical Sciences, Tehran, Iran

**Keywords:** Radiography, Ultrasonography, Standards, Management

## Abstract

**Background:**

Maintenance of imaging equipment is a very important part of the management of all medical imaging centers.

**Objectives:**

To assess the oldness and capacity of radiography and ultrasound equipment in Tehran University of Medical Sciences.

**Materials and Methods:**

The study was performed in 16 hospitals, 4 faculties and three healthcare centers of Tehran University of Medical Sciences. We evaluated all the X-ray equipment (including the simple plain and dental, panorex, mammography, fluoroscopy and C-arm X-Ray devices) and also simple and Doppler ultrasound machines in terms of the type and usage of the device, production year, quantity of utilization, location, brand and current condition.

**Results:**

Among fixed X-ray systems, 15 were currently in use, two were junk, two were damaged, and one was not utilized. The mean (SD) of the usage of these was 2151 (2230) cliché/month, and the mean (SD) of the oldness was 16.9 (13.6) years. The oldness of radiography equipment in our study was more than 20 years in 16, between 11 and 20 in 46, and less than 10 years in 76 devices. The mean (SD) usage (patients/month) of simple and color Doppler devices were 234.1 (365.2) and 597.5 (505.3), respectively. The oldness of ultrasonography equipment in our study was more than 11 years in 12 and less than 10 years in 55 devices. We found that 22 (15.9%) of the radiography systems and two (3%) of the ultrasonography systems had been used for more than 20 years.

**Conclusion:**

Radiology equipment in Tehran University of Medical Sciences have potential capacity, but they need repair, and better maintenance and management and application of standards for the imaging system needs organized supervisory mechanisms.

## 1. Background

Radiology wards are the very important and expensive parts of every medical center and presence of their disturbances may cause irretrievable damage to the patients and healthcare providers (1,2). It has been estimated that at least 25% of the patients need diagnostic imaging for their treatment (3) and more than 80% of those who refer to hospitals need diagnostic imaging. The most prevalent imaging request is X-ray. It is the second most common paraclinical assessment after laboratory studies. Simple X-ray and ultrasonography cover more than 90% of all imaging requirements (3, 4).

Progressive development in diagnostic imaging systems and costs during the past two decades indicate that utilization of imaging technology has increased in the world. Interestingly, new advances in this field can substitute previous technologies resulting in less interventions, radiation dose, cost and time.

Assessing the quality and obsolescence of radiological equipment and comparing them with the reliable international standards has a vital role in the quality assurance in image taking as well as providing care. Over-usage of some devices makes them damaged and subsequently imposes high-cost for repair. On the other hand, sub-optimal usage of some devices can cause economical and non-economical harm for patients and healthcare systems. 

## 2. Objectives

The aim of this study was to assess the oldness and capacity of radiography and ultrasound equipment in Tehran University of Medical Sciences.

## 3. Materials and Methods

The study covered all hospitals and health care institutions affiliated to Tehran University of Medical Sciences and included 16 hospitals, 4 faculties and three healthcare centers. We evaluated all X-ray equipment (fixed and portable), including simple plain and dental, panorex, mammography, fluoroscopy and C-arm X-ray devices and also simple and Doppler ultrasound machines. All data included the type and usage of the device, production year, quantity of utilization, location, brand, current condition, and maintenance of the devices.

## 4. Results

There were 138 X-ray radiology devices in 21 centers (Table 1). 

**Table 1.** Number of X-Ray Devices

Type of X-Ray Device	Number
**Fixed X-Ray**	20
**Portable**	60
**Dental**	15
**Panorex**	3
**Mammography**	6
**Fluoroscopy**	19
**C-arm**	15

Among fixed X-ray systems, 15 were currently in use, two were junk, two were damaged, and one was not utilized. In addition to these fixed devices, 15 were located in radiology and 5 were located in the other wards. The mean (SD) of their usage was 2151 (2230) cliché/month, and the mean (SD) of the oldness was 16.9 (13.6) years. Among radiography devices, 35 (25.4%) take less than 60 cliché/month, and 9 devices (6.5%) depict more than 3000 cliché monthly.

The oldness of radiography equipment was more than 20 years in 16, between 11 and 20 in 46 and less than 10 years in 76 devices. The mean of the cliché and oldness of the equipment are shown in Table 2. 

**Table 2.** The Mean of the Cliché and Oldness of the Equipment

Type of X-Ray Equipment	Mean (SD) of Taken Cliché (number/Month)	Mean (SD) of Oldness (year)
**Portable**	127 (190)	12.3 (7.6)
**Dental**	1342 (1016)	10.1 (7.2)
**Panorex**	950 (828)	8.6 (8.6)
**Mammography**	165 (168)	6.6 (3.8)
**Fluoroscopy**	778 (1118)	13.2 (12.8)
**C-arm**	319 (311)	10.2 (8.3)

Abbreviation: SD; standard deviation

Abbreviation: SD; standard deviation

Among portable systems, 50 were currently in use and functional, six were damaged and four were junk. There was 30 simple and 37 color Doppler ultrasounds in our centers. Among simple ultrasounds, 25 were currently in use and functional, two were currently in use and inoperative, two were damaged, and one was junk. The mean (SD) usage (patients/month) of simple and Doppler devices were 234.1 (365.2), and 597.5 (505.3), respectively. The mean (SD) oldness of simple and Doppler devices were 8.9 (5.3) years, and 4.9 (4.3) years, respectively. The oldness of ultrasonography equipment was more than 11 years in 12 and less than 10 years in 55 devices. We found that 22 (15.9%) of the radiography systems and two (3%) of the sonography systems had been used for more than 20 years. There were 16 (11.6%) radiography and 5 (7.5%) sonography devices that were out of order. Twelve (8.7%) of the radiography and 3 (4.5%) of the ultrasonography equipment were junk. Although 20 ultrasound devices (29.9%) gave service to more than 20 patients a day, 12 systems gave service to less than two patients.

## 5. Discussion

Equipment obsolescence; overuse and misuse of the equipment, are among factors that may cause defects in the quality of providing healthcare (5). These defects may be resolved by modernization of the devices as well as better supervision of the medical imaging services. The most important point of this study was the oldness of our equipment that needs rapid decision and renovation. The oldness of 15.9% of the simple X-ray systems in our study were more than 20 years that emphasizes the need for planning and a time-table to provide the expenses for their replacement with newer and updated equipments. We found that the mean age of fluoroscopy equipment were more than the other devices (Table 2). This equipment are known to emit the highest dose of radiation, and this is the reason why we are extremely concerned with the age of this system that did not even meet the less stringent criteria of less than 10 years of age. Rahimi and colleagues, in Mazandaran university, found that among 30 radiology equipments in 15 radiology centers, five devices were damaged and the mean age of their devices was 10 years (range: 10-30) (6) indicating that oldness of radiology devices is a general problem in our country and necessitates awareness of authorities and decision making for renovation as soon as possible. In this study, we found that in Tehran University of Medical Sciences, radiology devices are from different brands. This suggests that the authorities did not notice this point and some equipment were purchased from invalid companies. We found that 16 (11.6%) of the radiographic and 5 (7.5%) of the ultrasonography equipmens were damaged indicating that a large amount of our capital is useless and needs rapid verification and repair. Among devices, 12 (8.7%) of the radiographic and 3 (4.5%) of the ultrasonography ones were junk that needed discharge and replacement; besides it seems that repairing these junk systems are not cost-effective. Another important point in our centers was diversity in providing service that was reflected in the mean number of cliché and ultrasonography. This suggests better management for optimal application in different centers according to the standards of each device. 

In conclusion, radiology equipment in Tehran university of Medical Sciences have potential capacity, but they need repair, and better management and application of standards for the imaging system needs systematic and organized supervisory mechanisms.
